# Validation of SEPI in German—A German Translation of the Sun Exposure and Protection Index

**DOI:** 10.3390/ijerph17176172

**Published:** 2020-08-25

**Authors:** Elias Karlsson, Inga-Marie Hübner, Daniela Haluza, Magnus Falk

**Affiliations:** 1 Department of Medicine, Health and Caring Sciences, Linköping University, SE-581 83 Linköping, Sweden; elika216@student.liu.se (E.K.); magnus.falk@liu.se (M.F.); 2 Association of Dermatological Prevention [ADP], 20457 Hamburg, Germany; 3 Department of Environmental Health, Medical University of Vienna, 1090 Vienna, Austria; daniela.haluza@meduniwien.ac.at

**Keywords:** sunlight, ultraviolet radiation, skin cancer, questionnaire, prevention

## Abstract

The Sun Exposure and Protection Index (SEPI) is a brief instrument for scoring of sun exposure habits and propensity to increase sun protection, previously validated in English and in Swedish, as well as in two different outdoor sun intensity environments (Australia and Northern Europe). The aim of the present study was to study reliability and validity of a German translated version of the SEPI to be used in German-speaking populations. Data was collected at University of Flensburg and at Hamburg University of Applied Sciences from November 2018 to April 2019. Participants (n = 205) filled out the SEPI and also a selection of corresponding questions from the Austrian Vienna UV Questionnaire in German. After three weeks, the participants filled out the SEPI once again in order to assess test–retest stability. Of the 205 participants completing the baseline questionnaire, 135 participants completed it once again after three weeks. Internal consistency, by Cronbach’s alpha, for the baseline responses was 0.70 (95% C.I: 0.63–0.76) for SEPI part 1 (sun exposure habits) and 0.72 (95% C.I: 0.66–0.78) for part 2 (propensity to increase sun protection). Test–retest stability was high, with weighted Kappa >0.6 for all items but one, and the instrument correlated well with the previously validated German-language UV Skin Risk Survey Questionnaire. In conclusion, the German version of SEPI can reliably be used for mapping of individual sun exposure patterns.

## 1. Introduction

The different forms of skin cancer are the most common malignant tumour entities [1,2], with a clear majority of cases occurring in Caucasian populations worldwide [3]. Skin cancer derives either from keratinocytes (basal cell carcinoma, BCC, and squamous cell carcinoma, SCC) or from melanocytes (malignant melanoma, MM), of which the latter is the most lethal, especially if not detected at an early stage. However, although rarely lethal, but on the other hand considerably more common and frequently occurring on the head and face, keratinocytic skin cancers are associated with voluminous patient suffering and healthcare costs due to necessary surgical and cosmetic interventions [1]. According to a recent extensive systematic review, the global burden of cancer, reflected in DALYs (Disability-Adjusted Life-Years), has during the last decade grown to take the second highest position, to date only exceeded by the burden of cardiovascular disease [4].

In all skin cancers, exposure to ultraviolet radiation (UVR) is the main environmental risk factor and an initiator of carcinogenic mutations [5,6,7,8]. Thus, skin cancer is often referred to as highly preventable, as primary preventive measures can be directed at promoting accurate sun protection to individuals, or groups of individuals, with the highest risk due to phenotypic, environmental, and behavioural factors [9,10,11,12,13,14,15,16,17]. In order to be able to do so, there is a need for valid and reliable tools to measure sun exposure habits and sun protection behaviour, both in order to identify individuals with a risky behaviour with regard to skin cancer, as well as to be able to communicate individually tailored sun protection advice [18,19,20]. For this purpose, the Sun Exposure and Protection Index (SEPI) has previously been developed and validated in English and in Swedish, as well as in two opposing UVR environments (Australia and Northern Europe) [21], and since then used as a measure in both epidemiologic and experimental studies [19,22,23]. It is composed of two parts; part 1 addresses present sun exposure habits, and part 2 the propensity to increase sun protection. Completing it results in a score for each part; in part 1 (0–32 points), a high score reflects a high UVR risk exposure, with regard to skin cancer, and in part 2 (0–20 points), a high score reflects a low propensity to increase sun protection. Thus, the instrument has the capacity to identify those individuals with the most pronounced risk behaviour in the sun, as well as their readiness (and consequently their likelihood) to undertake enhanced sun protective actions. In comparison to many other questionnaires investigating sun exposure habits, the SEPI has the advantage of being brief, taking only a few minutes to complete, making it feasible in the clinical situation.

Since the SEPI has not previously been available in the German language, the aim of the present study was to investigate the validity and reliability of a German translation of the SEPI questionnaire, for use in German-speaking populations (Supplementary Materials).

## 2. Materials and Methods

### 2.1. Translation Procedure

SEPI was translated into German by a translation-back translation method [24], where in a first step, two blinded, native German speakers translated the original English version of the instrument into German, independently. In a second step, the translated German version was translated back into English by two different native German speakers unfamiliar with the English original version. The resulting back translations were compared for accuracy, in terms of agreement with one another and with the original. From this process, no revisions needed to be done since the back translations yielded sufficient level of agreement (close to identical, as evaluated by the authors qualitatively), indicating that the questionnaire contents were preserved throughout the translation process. In a final third step, two further native German speakers independently pilot tested the tool and evaluated it from a contextual perspective, to ensure general comprehensibility, face validity and content validity. No revisions were needed at this stage either.

### 2.2. Study Population

The study population consisted of first- and third-semester bachelor students of health sciences at the University of Flensburg and at Hamburg University of Applied Sciences. Exclusion criteria were age <18 years and inability to fill out the questionnaire or identification code used for the follow-up procedure (see below). Participants were recruited with the consent of the tutor by the scientific team during the lectures who also provided them with short oral information and a written study information sheet, including a statement that participation was voluntary and not linked to any course achievements, and no incentives or reimbursement were given for participation. The study was conducted following the ethical standards laid down in the Declaration of Helsinki. An additional ethical approval was not mandatory according to the university and authority guidelines.

### 2.3. Data Collection

Together with the study information, the participants were handed a questionnaire containing the two parts of the SEPI and the corresponding question most closely addressing the same aspect of sun exposure or protection, from the already validated, somewhat more extensive, Austrian UVSRS (UV Skin Risk Survey) questionnaire. The latter was developed for a previous study [25,26,27,28], is also in German and was considered to be suitable for assessment of criterion validity of the German SEPI version. Like the SEPI, the UVSRS questionnaire is based on ordinal response alternatives (Likert scales), although, unlike the strictly five-grade SEPI, the number varied somewhat between questions (see Table 3). To assess test stability over time, a test–retest procedure was performed in a second survey wave three weeks later. Since the study relied on comparing answers from the same subjects at these two different occasions, with guaranteed confidentiality, pseudonymised, unique identification codes were developed. The participants created a unique code from the letters in prespecified positions in their first and last names in combination with prespecified number positions in their date of birth. This way, the identification code would be identical both times while not providing sufficient information to determine actual identity, since no information about birth month or other letters in any name was provided. As demographic data, gender and age were asked for, the latter in categorical terms (18–29 years, 30–39 years, 40–49 years, 50–59 years, ≥60 years) in order to secure confidentiality.

### 2.4. Statistical Analyses

For analysis of internal consistency, Cronbach’s alpha was determined for both parts of SEPI from the test responses. Values of alpha ≥0.7 were considered to indicate acceptable internal consistency [29]. Test–retest stability was measured by determining the agreement for each item at baseline and after three weeks, using Cohen’s weighted Kappa analysis, with its 95% confidence intervals retrieved from the standard error and z-distribution [30]. A Kappa value >0.4 is generally viewed as moderate agreement, >0.6 as substantial and >0.8 as almost perfect agreement [31]. Criterion validity was measured by calculating Spearman’s rho when the participant responses to each SEPI question were compared with the responses to corresponding items from the UVSRS instrument. In this case, Kappa analysis was not used since the SEPI and UVSRS questions differed in number of response alternatives. Only the responses from the first of the two SEPI response occasions were used for the comparison. A Spearman’s rho value of ≥0.7 was considered as good correlation. The 95% confidence intervals for rho were based on 1000 bootstrap samples. For all statistical analysis, SPSS 26.0 software was used (SPSS Inc., Chicago, IL, USA).

## 3. Results

The questionnaire containing both SEPI and the corresponding relevant question items from the UVSRS questionnaire was completed by 205 students (79% female, 21% male, 93% aged 18–29 years, 7% aged >29 years). Of these, 135 participants also responded to the follow-up questionnaire (containing only SEPI part 1 to be filled out a second time), for which, however, 22 had provided an identity coding not matching any of those from the corresponding first response occasion, disabling test–retest comparison for these individuals. This left 113 participants for test–retest analyses (80% female, 20% male, 92% aged 19–29 years, 8% aged >29 years). The mean SEPI score did not differ significantly between responders and nonresponders (15.4 versus 15.2 points, respectively, for SEPI part 1, and 9.6 points in both cases for SEPI part 2).

### 3.1. Internal Consistency

Internal consistency of the two parts of SEPI is shown in Table 1, for each of the individual questions also displaying the outcome if the question were to be excluded. For both parts of the scale, Cronbach’s alpha was >0.7, with a somewhat higher value for SEPI part 2. Sunscreen use was the only item rendering a stronger internal consistency if excluded from the scale, indicating an individual contribution in an opposite direction than the other items.

### 3.2. Test–Retest Stability

The questionnaire stability over time, based on weighted Kappa analysis between SEPI responses at baseline and after three weeks follow-up, is shown in Table 2. In the Kappa analysis, seeking the shade was the only question item with a value below 0.6.

### 3.3. Criterion Validity

Criterion validity for the two parts of the SEPI, as tested by comparison with the UVSRS questionnaire, is shown in Table 3 by means of Spearman’s rho assessed for each SEPI question item and a corresponding item from UVSRS. For most of the questions, the rho value was higher than or only slightly below 0.7 (considered as acceptable correlation). The lowest value (0.48) was found for “vacational sun exposure”.

## 4. Discussion

SEPI has already been validated for usage as a screening tool in Swedish and English [21]. In the present study, it has been tested also in German. Overall, the results suggest the German version of the SEPI to be both valid and reliable, opening for it to be used in relevant clinical and research-related situations.

Internal consistency for both SEPI parts, as measured by Cronbach’s alpha, was acceptable and on level with (or even slightly higher than) the corresponding outcome found in the original validation study in English and Swedish [21] (0.7 compared to 0.69). As in that study, sunscreen use was the item that contributed least to the score, results showing an alpha value that would be somewhat increased if the question were to be deleted. As already pointed out in the original article, this can probably be explained by the well-known “sunscreen paradox”, that is, a tendency for many of those applying a sunscreen to do so in order to enable a longer stay in the sun, rather than to reduce sun exposure [32,33]. However, in contrast to the findings in the English/Swedish validation study, the described phenomenon was in fact less pronounced for the German version.

Criterion validity of the SEPI likewise proved good, as shown by the overall acceptable, and in some cases, somewhat weaker, correlations between their ingoing question components and the corresponding UVSRS items. The weaker correlations for these question items are likely to reflect that the items are not entirely equal in construct; for example, the SEPI question exploring vacation sun exposure investigates the frequency of vacations in the sun, whilst the closest corresponding UVSRS item addresses the sum of total outdoor sun exposure time [25]. Likewise, there are no questions regarding propensity to change behaviour in the UVSRS, but there were nonetheless good correlations with the items from SEPI part 2. Regarding test stability over time, the test–retest assessment showed “substantial” or “almost perfect” agreement according to kappa analysis, for all except one item, indicating the SEPI to be reproducible and useful (e.g., for follow-up of a given sun protection directed intervention). The results are on level with, or for several items somewhat higher than, what has been found in previous studies exploring reproducibility of self-reported measures of sun exposure and protection [34,35].

Besides the moderate sample size and the relatively high proportion of dropouts in the test–retest part, a limitation of this study was the relatively young age of respondents. However, the original SEPI validation study was performed in a university student population as well, but also in a primary care population, thus with a considerably greater heterogeneity with regard to age and educational level. The main issue in the present study was not whether the SEPI, as an instrument, was valid per se, since this has already been stated, but to investigate if it sustained its validity when translated into German and applied in a German-speaking population. We assume that younger people are yet to be considered a specifically important risk group in terms of having in general more risky sun habits than other age groups, and at the same time being in a stage of life when reduced UV exposure would have the highest long-term preventive value with regard to skin cancer, with emphasis on melanoma [20,28]. A general limitation of the SEPI, like many other behaviour-related questionnaires, is that it is based on self-reported measures, and has not been validated against any objective measure such as provision of an individual UV meter to assess the degree of actual UV exposure, an area of potential future instrument improvement in terms of validation.

Compared to the more extensive UVSRS questionnaire [21], SEPI is shorter and has briefer questions. It is also delimitated to behaviour, whereas the UVSRS also includes a few additional aspects, such as knowledge of information sources on UV risks and protection. Also, SEPI contains the section (part 2) exploring propensity to increase sun protection, which together with its short-format concept makes it suitable in clinical situations where it might be relevant to communicate sun protection advice, such as patients having a skin check, situations where shortness of time is often a limiting factor. Using a prefilled SEPI prior to the skin check (e.g., in the waiting room or at home) may constitute a valuable information source for the physician to be able to target important advice to the patients in most need of it (according to SEPI part 1), as well as to those most likely to actually comply (according to SEPI part 2). In an Australian population, Cargill et al. tested the reliability of brief questions against diary recorded sun exposure and found it to correlate well [36]. In Germany, as in many other countries, increasing skin cancer incidence [37,38] warrants a subsequent intensified need for skin cancer to be managed in primary care. Since the prevention of skin cancer hinges upon UV avoidance, broad guidance in assessing sun exposure habits and communicating sun protection advice is likely to be needed to slow down the development [39,40,41]. In this respect, SEPI is a brief instrument that can be used in routine patient–doctor consultations. Especially the use in the German nationwide skin cancer screening program that includes consulting on sun protection behaviour should be emphasised.

## 5. Conclusions

In conclusion, this study showed the German translation of SEPI to be both valid and reliable, to be used as a clinical risk assessment tool for dermatologists and general practitioners in German-speaking countries, with regard to skin cancer, as well as being a measurement tool in research studies addressing sun exposure, for example, to evaluate the effect of an intervention to promote sun protective habits.

## Figures and Tables

**Table 1:** Internal consistency of SEPI part 1, in terms of Cronbach’s alpha, and its 95% confidence intervals, displayed for the total score and with deletion of each of the individual items.

	Cronbach’s Alpha (n = 205)	95% C.I.
Value for SEPI part 1 total score	0.70	0.63–0.76
Value after deletion of a single item, as follows		
1. How often do you sunbathe with the intention to get tanned?	0.63	0.55–0.70
2. How many times have you been sunburnt [redness and smarting pain] during the last 12 months?	0.70	0.64–0.76
3. How long do you usually stay in the sun [on average] between 11 am and 3 pm, on a typical day off?	0.66	0.58–0.72
4. How often do you take a holiday with the intention of spending more time in the sun?	0.67	0.59–0.73
5. When in the sun, how often do you use sunscreen?	0.74	0.68–0.79
6. When in the sun, how often do you use covering clothes for protection?	0.63	0.55–0.70
7. When in the sun, how often do you use a sun hat or cap for sun protection?	0.70	0.63–0.76
8. How often do you stay indoors or in the shade in order to protect yourself from the sun?	0.64	0.56–0.71
Value for SEPI part 2 total score	0.72	0.66–0.78
Value after deletion of a single item, as follows		
1. Attitude towards the individual’s sunbathing	0.72	0.65–0.78
2. Attitude towards sunscreen usage	0.73	0.67–0.79
3. Attitude towards usage of covering clothes	0.60	0.51–0.69
4. Attitude towards usage of sun hat or cap	0.70	0.62–0.76
5. Attitude towards seeking shade	0.59	0.49–0.67

**Table 2:** Stability over time, by Cohen’s weighted kappa, between the SEPI question items at baseline and at follow-up, and its 95% confidence intervals.

Item	Weighted Kappa (n = 113)	95% C.I.
SEPI part 1		
1. How often do you sunbathe with the intention to get tanned?	0.68	0.59–0.80
2. How many times have you been sunburnt [redness and smarting pain] during the last 12 months?	0.72	0.62–0.83
3. How long do you usually stay in the sun [on average] between 11 am and 3 pm, on a typical day off?	0.57	0.44–0.69
4. How often do you take a holiday with the intention of spending more time in the sun?	0.75	0.66–0.83
5. When in the sun, how often do you use sunscreen?	0.67	0.58–0.77
6. When in the sun, how often do you use covering clothes for protection?	0.60	0.52–0.72
7. When in the sun, how often do you use a sun hat or cap for sun protection?	0.62	0.52–0.72
8. How often do you stay indoors or in the shade in order to protect yourself from the sun?	0.52	0.40–0.65
SEPI part 2		
1. Attitude towards the individual’s sunbathing	0.80	0.66–0.86
2. Attitude towards sunscreen usage	0.66	0.55–0.77
3. Attitude towards usage of covering clothes	0.69	0.58–0.78
4. Attitude towards usage of sun hat or cap	0.71	0.62–0.81
5. Attitude towards seeking shade	0.61	0.50–0.71

**Table 3:** Correlation between SEPI question responses and the associated question in the UVSRS questionnaire (in italics), by Spearman’s rho, and its 95% confidence intervals. The table also displays the number of response alternatives for each of the UVSRS questions.

SEPI Question and Associated UVSRS Questions		Spearman’s Rho (n = 205)	95% C.I.
**SEPI Part 1**	Associated UVSRS question		
1. Intentional tanning	Please think about last year: How many days did you sunbathe outside? (5 response alternatives)	0.67	0.59–0.74
2. Occasions with sunburn	How often have you had sunburn (painful reddening of the skin) in the last year? (5 response alternatives)	0.87	0.83–0.90
3. Duration of stay in the sun	How long did your sunbath last on average? (4 response alternatives)	0.54	0.44–0.63
4. Vacational sun exposure	Please think about last year: How many days did you sunbathe outside? (5 response alternatives)	0.48	0.37–0.58
5. Sunscreen use	How often do you use the following methods to protect yourself from the sun? - I use sunscreen with a sun protection factor of at least SPF 15. (5 response alternatives)	0.82	0.77–0.86
6. Clothes for sun protection	How often do you use the following methods to protect yourself from the sun? - I wear protective clothes. (5 response alternatives)	0.76	0.70–0.81
7. Hat or cap for sun protection	How often do you use the following methods to protect yourself from the sun? - I wear protective headgear. (5 response alternatives)	0.86	0.82–0.89
8. Staying indoors or in the shade	How often do you use the following methods to protect yourself from the sun? - I stay in the shade. (5 response alternatives)	0.62	0.53–0.70
**SEPI Part 2**			
1. Sunbathing	Please think about last year: How many days did you sunbathe outside? (5 response alternatives)	0.60	0.50–0.68
2. Sunscreens	How often do you use the following methods to protect yourself from the sun? - I use sunscreen with a sun protection factor of at least SPF 15. (5 response alternatives)	0.67	0.59–0.74
3. Covering clothes	How often do you use the following methods to protect yourself from the sun? - I wear protective clothes. (5 response alternatives)	0.74	0.67–0.80
4. Sun hat or cap	How often do you use the following methods to protect yourself from the sun? - I wear protective headgear. (5 response alternatives)	0.77	0.71–0.82
5. The shade	How often do you use the following methods to protect yourself from the sun? - I stay in the shade. (5 response alternatives)	0.64	0.55–0.71

## Supplementary Materials

The following are available online at https://www.mdpi.com/1660-4601/17/17/6172/s1.
