# Supplementary material for: Validation of SEPI in German—A German Translation of the Sun Exposure and Protection Index
Source: Int J Environ Res Public Health. 2020 Aug 25;17(17):6172. doi: 10.3390/ijerph17176172 (PMC7504206; doi:10.3390/ijerph17176172)
Supplement: Supplementary file 1 [file ijerph-17-06172-s001.pdf]

International Journal of
Environmental Research and Public Health

MDPI

Supplementary Materials: Validation of SEPI in German—A German Translation of the Sun Exposure and Protection Index

SEPI in English and in German

Sun Exposure and Protection Index (SEPI)
– Part I –

1. How often do you sunbathe with the intention to get tanned?

   0 ☐ Never
   1 ☐ Seldom
   2 ☐ Occasionally
   3 ☐ Often
   4 ☐ Always

2. How many times have you been sunburnt (redness and smarting pain) during the last 12 months?

   0 ☐ None
   1 ☐ 1-2 times
   2 ☐ 3-5 times
   3 ☐ 6-10 times
   4 ☐ More than 10 times

3. How long do you usually stay in the sun (on average), between 11 am and 3 pm, on a typical day-off?

   0 ☐ < 30 min
   1 ☐ 30 min – 1 hour
   2 ☐ 1-2 hours
   3 ☐ 2-3 hours
   4 ☐ > 3 hours

4. How often do you take a holiday with the intention of spending more time in the sun?

   0 ☐ Never
   1 ☐ Seldom
   2 ☐ 1-2 weeks a year
   3 ☐ 3-5 weeks a year
   4 ☐ > 5 weeks a year

5. When in the sun, how often do you use sunscreens?

   0 ☐ Always
   1 ☐ Often
   2 ☐ Occasionally
   3 ☐ Seldom
   4 ☐ Never

6. When in the sun, how often do you use covering clothes for sun protection?

   0 ☐ Always
   1 ☐ Often
   2 ☐ Occasionally
   3 ☐ Seldom
   4 ☐ Never

7. When in the sun, how often do you use a sun hat or cap for sun protection?

   0 ☐ Always
   1 ☐ Often
   2 ☐ Occasionally
   3 ☐ Seldom
   4 ☐ Never

8. How often do you stay indoors or in the shade in order to protect yourself from the sun?

   0 ☐ Always
   1 ☐ Often
   2 ☐ Occasionally
   3 ☐ Seldom
   4 ☐ Never

– Part II –

Choose for each item which statement is most consistent with your own state:

1. Sunbathing:
   - 4 ☐ I have never thought of giving up sunbathing.
   - 3 ☐ I could think of giving up sunbathing.
   - 2 ☐ I intend to give up sunbathing.
   - 1 ☐ I have recently given up sunbathing .
   - 0 ☐ I have for a long time avoided sunbathing

2. Sunscreens:
   - 4 ☐ I have never thought of using sunscreens.
   - 3 ☐ I could think of using sunscreens.
   - 2 ☐ I intend to start using sunscreens.
   - 1 ☐ I have started to use sunscreens.
   - 0 ☐ I have for a long time used sunscreens.

3. Covering clothes:
   - 4 ☐ I have never thought of using covering clothes for sun protection.
   - 3 ☐ I could think of using covering clothes for sun protection.
   - 2 ☐ I intend to start using covering clothes for sun protection.
   - 1 ☐ I have started to use covering clothes for sun protection.
   - 0 ☐ I have for a long time used covering clothes for sun protection.

4. Sun hat or cap:
   - 4 ☐ I have never thought of using a sun hat or cap for sun protection.
   - 3 ☐ I could think of using a sun hat or cap for sun protection.
   - 2 ☐ I intend to start using a sun hat or cap for sun protection.
   - 1 ☐ I have started to use a sun hat or cap for sun protection.
   - 0 ☐ I have for a long time used a sun hat or cap for sun protection.

5. The shade:
   - 4 ☐ I have never thought of trying to stay in the shade during the hours of strongest sun light.
   - 3 ☐ I could think of trying to stay in the shade during the hours of strongest sun light.
   - 2 ☐ I intend to start trying to stay in the shade during the hours of strongest sun light.
   - 1 ☐ I have started trying to stay in the shade during the hours of strongest sun light.
   - 0 ☐ I have for a long time tried to stay in the shade during the hours of strongest sun light.

– Scoring instructions –

SEPI part I: For each question, the given response score (0-4 p), should be added together in a total score (0-32 p), reflecting *increasing UV risk exposure.*

SEPI part II: For each question, the given response score (0-4 p), should be added together in a total score (0-20 p), reflecting *decreasing propensity to increase sun protection.*

**SEPI - part I** score:	**SEPI - part** score:

LINKÖPING UNIVERSITY

Karolinska Institutet

QIMR Berghofer Medical Research Institute

Sun Exposure and Protection Index (SEPI)
– Teil I –

1. Wie oft sonnenbaden Sie mit der Absicht braun zu werden?
   - 0 ☐ Nie
   - 1 ☐ Selten
   - 2 ☐ Manchmal
   - 3 ☐ Oft
   - 4 ☐ Immer

2. Wie oft haben Sie in den letzten 12 Monaten Sonnenbrand gehabt (Rötung und Schmerzen)?
   - 0 ☐ Nie
   - 1 ☐ 1-2 Mal
   - 2 ☐ 3-5 Mal
   - 3 ☐ 6-10 Mal
   - 4 ☐ Mehr als 10 Mal

3. Wie lange bleiben Sie normalerweise in der Sonne (im Durchschnitt), zwischen 11 und 15 Uhr, an einem typischen freien Tag?
   - 0 ☐ < 30 Minuten
   - 1 ☐ 30 Minuten – 1 Stunde
   - 2 ☐ 1-2 Stunden
   - 3 ☐ 2-3 Stunden
   - 4 ☐ > 3 Stunden

4. Wie oft machen Sie Ferien mit der Absicht mehr Zeit in der Sonne zu verbringen??
   - 0 ☐ Nie
   - 1 ☐ Selten
   - 2 ☐ 1-2 Wochen pro Jahr
   - 3 ☐ 3-5 Wochen pro Jahr
   - 4 ☐ > 5 Wochen pro Jahr

5. Wenn Sie in der Sonne sind, wie oft benutzen Sie Sonnencreme?
   - 0 ☐ Immer
   - 1 ☐ Oft
   - 2 ☐ Manchmal
   - 3 ☐ Selten
   - 4 ☐ Nie

6. Wenn Sie in der Sonne sind, wie oft benutzen Sie bedeckende Kleidung als Sonnenschutz?
   - 0 ☐ Immer
   - 1 ☐ Oft
   - 2 ☐ Manchmal
   - 3 ☐ Selten
   - 4 ☐ Nie

7. Wenn Sie in der Sonne sind, wie oft benutzen Sie einen Sonnenhut oder eine Kappe als Sonnenschutz?
   - 0 ☐ Immer
   - 1 ☐ Oft
   - 2 ☐ Manchmal
   - 3 ☐ Selten
   - 4 ☐ Nie

8. Wie oft bleiben Sie drinnen oder im Schatten, um sich vor der Sonne zu schützen?
   - 0 ☐ Immer
   - 1 ☐ Oft
   - 2 ☐ Manchmal
   - 3 ☐ Selten
   - 4 ☐ Nie

– Teil II –

Wählen Sie bei jeden Punkt, welche Aussage am Besten auf Ihre eigene Lage zutrifft:

1. Sonnenbaden:

4 ☐ Ich habe nie daran gedacht das Sonnenbaden aufzugeben.
3 ☐ Ich könnte mir vorstellen das Sonnenbaden aufzugeben.
2 ☐ Ich habe die Absicht das Sonnenbaden aufzugeben.
1 ☐ Ich habe vor Kurzem das Sonnenbaden aufgegeben.
0 ☐ Ich meide das Sonnenbaden seit Langem.

2. Sonnencreme:

4 ☐ Ich habe nie daran gedacht Sonnencreme zu benutzen.
3 ☐ Ich könnte mir vorstellen Sonnencreme zu benutzen.
2 ☐ Ich habe die Absicht Sonnencreme zu benutzen.
1 ☐ Ich habe angefangen Sonnencreme zu benutzen.
0 ☐ Ich benutze Sonnencreme seit Langem.

3. Bedeckende Kleidung:

4 ☐ Ich habe nie daran gedacht bedeckende Kleidung als Sonnenschutz zu benutzen.
3 ☐ Ich könnte mir vorstellen bedeckende Kleidung als Sonnenschutz zu benutzen.
2 ☐ Ich habe die Absicht bedeckende Kleidung als Sonnenschutz zu benutzen.
1 ☐ Ich habe angefangen bedeckende Kleidung als Sonnenschutz zu benutzen.
0 ☐ Ich benutze bedeckende Kleidung als Sonnenschutz seit Langem.

4. Sonnenhut oder Kappe:

4 ☐ Ich habe nie daran gedacht einen Sonnenhut oder eine Kappe als Sonnenschutz zu benutzen.
3 ☐ Ich könnte mir vorstellen einen Sonnenhut oder eine Kappe als Sonnenschutz zu benutzen.
2 ☐ Ich habe die Absicht einen Sonnenhut oder eine Kappe als Sonnenschutz zu benutzen.
1 ☐ Ich habe angefangen einen Sonnenhut oder eine Kappe als Sonnenschutz zu benutzen.
0 ☐ Ich benutze einen Sonnenhut oder eine Kappe als Sonnenschutz seit Langem.

5. Schatten:

4 ☐ Ich habe nie daran gedacht zu versuchen im Schatten zu bleiben während der Stunden mit dem stärksten Sonnenlicht.
3 ☐ Ich könnte mir vorstellen zu versuchen im Schatten zu bleiben während der Stunden mit dem stärksten Sonnenlicht.
2 ☐ Ich habe die Absicht zu versuchen im Schatten zu bleiben während der Stunden mit dem stärksten Sonnenlicht.
1 ☐ Ich habe angefangen zu versuchen im Schatten zu bleiben während der Stunden mit dem stärksten Sonnenlicht.
0 ☐ Ich versuche seit Langem im Schatten zu bleiben während der Stunden mit dem stärksten Sonnenlicht.

LINKÖPING UNIVERSITY
Karolinska Institutet
QIMR Berghofer Medical Research Institute

ARBEITSGEMEINSCHAFT DERMATOLOGISCHE PRÄVENTION
